# Supplementary material for: The T6SS-Dependent Effector Re78 of Rhizobium etli Mim1 Benefits Bacterial Competition
Source: Biology (Basel). 2023 May 4;12(5):678. doi: 10.3390/biology12050678 (PMC10215855; doi:10.3390/biology12050678)
Supplement: Supplementary file 1 [file biology-12-00678-s001.zip › biology-2334338-supplementary.pdf]

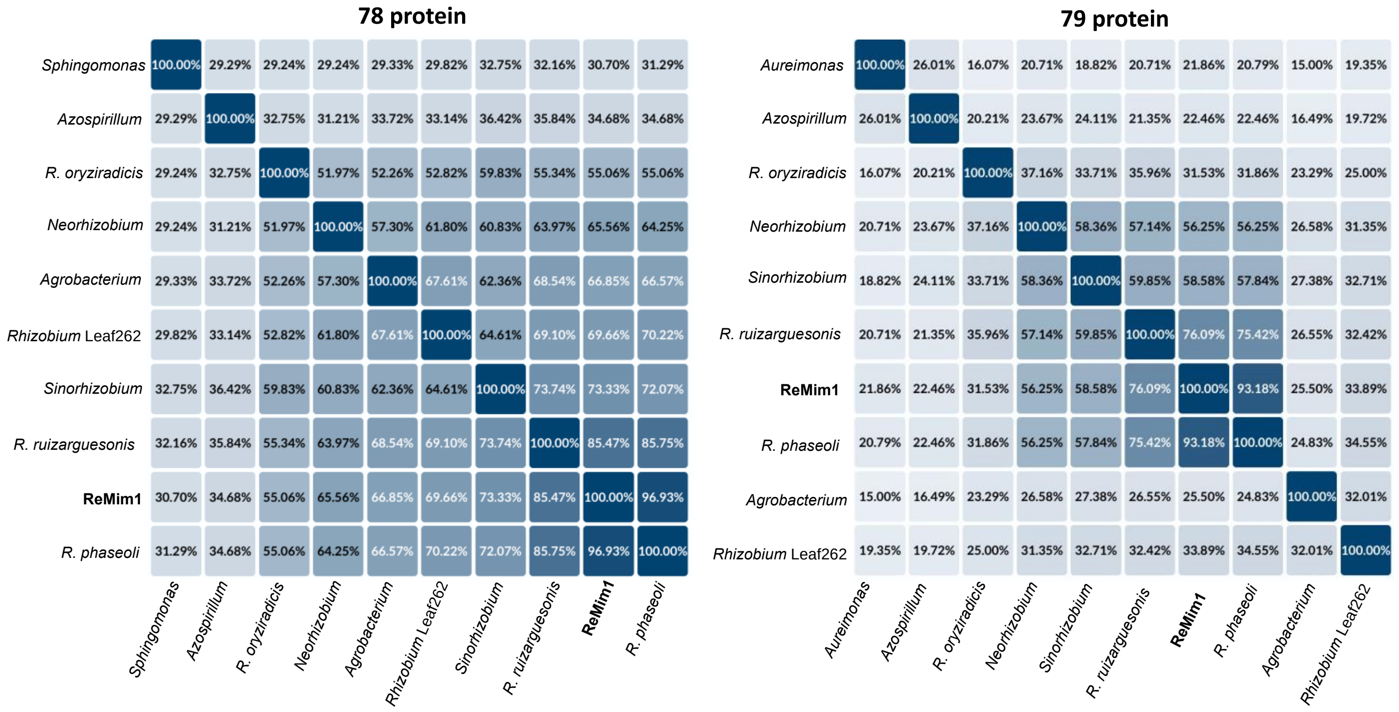


**Figure S1.** Heat map of the average amino acids identity (%) of proteins homologous to Re78 (REMIM1_PF00478) and Re79 (REMIM1_PF00479) of *Rhizobium etli* Mim1. The alignments were performed using ClustalW, version 1.2.4. of UniprotKB webserver.

(a)

```
R.etli Mim1 Re78 (AGS26143.1)      MIVSAATERL NDILVELPKY KSQWEANVSS PYVRAYDLAI DNFQKTTKAQ  50
Neorhizobium (CDN51818.1)          MIVASANQRL GDILVKLPKY KSQWELNVAS PYVRAYDAAV LSYNNEMKAQ  50
Sinorhizobium (APG87637.1)         MIVSSATDRL TDILVELPKY KSQWQSNVAN PYVRAYDLAV ENFQRTLKAQ  50
R.phaseoli (ANM08143.1)            --MSAATERL NDILVELPKY KSQWEANVSS PYVRAYDLAI DNFQKTTKAQ  48
Agrobacterium (WP_027675286.1)     --MSLATDRL NNILIELPKY KSQWQLNVAS PYVRAYDLAI DNYNKKMQEQ  48
R.ruizarguesonis (NKQ86457.1)      --MSAATDRL NDILIELPKY KSQWETNVAN PYVRAYDLAI DNYQKTTKAQ  48
R.oryziradicis (WP_075641707)      --MSSAKDRL TSIMDELDKK KSQWEVSVVS PYVRSYDFAY NNYQNTLKAQ  48
Rhizobium (WP_062451316)           --MSLAKERL NNILLELPKY ESQWESNIVL PYSRAYDLAI YNLNKVTKEQ  48
Sphingomonas (WP_156477649)        -----MADRL QTYRNQVSDA VDAWQANVCN PYIIAYNTAY KSYADVFKKQ  45
Azospirillum (WP_089286101)        -----MSDII RNYLDELEAK AIRWRIFCR- DYASAYHLAH DQYKATWAAQ  44

R.etli Mim1 Re78 (AGS26143.1)      EARDRMLAEL FVFSASILTG SIMMAAFATT SVRVLAGRAA LSVICNNNLN  100
Neorhizobium (CDN51818.1)          GERDRATAEM FVLAASILTG SVMMAAFATT SVRVMAGRAI VRVICNNNLN  100
Sinorhizobium (APG87637.1)         EARDKMTAEL FVFAASVLSG SVLMAAFATA SLRVLAGRAA LRVVCNNNLN  100
R.phaseoli (ANM08143.1)            EARDRMLAEL FVFSASILTG SIMMAAFATT SVRVLAGRAA LSVICNNNLN  98
Agrobacterium (WP_027675286.1)     AARDKMAAEL FVMSASILTG SVMMAVFATT SLRTLAGRAA LRVICNQNLN  98
R.ruizarguesonis (NKQ86457.1)      EARDRMLAEL FVFSASVLTG SIMMAAFATT SVRVLAGRTA LRVICNNNLN  98
R.oryziradicis (WP_075641707)      VARDKMGAEL LVFTAGVLSG SVLMAAFASS SLRVLAGRAL LRTICNNNLN  98
Rhizobium (WP_062451316)           EARDKLKAEL FVMSASILTG SVMMAAFATT SLRVLAGRAA LHVICNNNLN  98
Sphingomonas (WP_156477649)        DDSDKARAEL FVTAAAILPG SILMATAATS SLRVIANRTA VRLLAGRSTS  95
Azospirillum (WP_089286101)        EKSDKEKAEM IVLALSVASS TVLMAAFATN SLRVLASRAA LNVICNNNLN  94

R.etli Mim1 Re78 (AGS26143.1)      RTFNVMHAAA SNKVVMFALG GVLDEAKKLA NKQVTSAAEK LTSSAPVASA  150
Neorhizobium (CDN51818.1)          RTFNLMVAAN NNKAFMFALG GVLDEAKKVA NKQVTKLVEG LTSTGTQISA  150
Sinorhizobium (APG87637.1)         RTFDLMHAAA SNKAVMFALG GILDEAKKFA GKQITSAAEK LTSSAPIASS  150
R.phaseoli (ANM08143.1)            RTFNVMHAAA SNKVVMFALG GVLDEAKKLA NKQVTSAAEK LTSSAPVAAA  148
Agrobacterium (WP_027675286.1)     TTFNAFHAVS ESKVLMFALG GVLDEVKKLA GKHVTAAVEG LTSSDAIASA  148
R.ruizarguesonis (NKQ86457.1)      RTFDLMHAAA TSKPVMFALG GVLDEAKKLA NKQVTSAAEK LTSSAPIASA  148
R.oryziradicis (WP_075641707)      RTFDAVHAVT NNKAAMFALG SVLDKAKGIA GQHITAAVEN FTVRTSGIQS  148
Rhizobium (WP_062451316)           RTFEAMRAAS ANKALMFAFG SVLDEAKKLA GKQVTSAVEG LVSSASIASA  148
Sphingomonas (WP_156477649)        RALGVYNAVA SNATATFAIG KALDIVKDET GKAIKDSVVK TMSNTRDLLA  145
Azospirillum (WP_089286101)        RTFNALHLVS NNKAAMFALG AVMDAADAKA KKYIEEKAGT LTRSAPTPAV  144

R.etli Mim1 Re78 (AGS26143.1)      PTALNFKTRI DDFIAINHVC VHEFIRGVQQ DNTISEAGKS RLADLVGQTP  200
Neorhizobium (CDN51818.1)          PTSVNYESRL MDFIRVSHIC THSFIEGVKE DNSISETHKA QVADMAARIP  200
Sinorhizobium (APG87637.1)         PTAINFLTRI EDFINVNHIC VHEFIAGVRD DRAISETDKL KVADLVGKTP  200
R.phaseoli (ANM08143.1)            PTALNFKTRI DDFIAINHVC VHDFVRGVQH DNTISEAGKS RLADLVAQTP  198
Agrobacterium (WP_027675286.1)     PTALNFLTRM EDFINVNHIC VHNFVQGVRD DASISDANKT HIAELVGKTP  198
R.ruizarguesonis (NKQ86457.1)      PTALNFMTRI ADFIDTNHIC VHDFVRGVQQ DNSISEANKS RLADLVGKTP  198
R.oryziradicis (WP_075641707)      QTSVNFLTRL QDFVNVSFIA VHEFVTGVRN DNSIKESDKL KLADMVEATP  198
Rhizobium (WP_062451316)           PTGLNFESRM KTFVNDNHIC VHSFVQGVRD DGSLKDDDKM HIAELATKMP  198
Sphingomonas (WP_156477649)        TDPLNRDKQL NSWLINHKLL AYDAADAIER SRSMSERDKQ RAYDLLRAAP  195
Azospirillum (WP_089286101)        TNPAVLQNDL INYVESNQLY AMEVAFAIRD DKSLSEADRA ILVGSLKRAP  194

R.etli Mim1 Re78 (AGS26143.1)      FCNPPESRRV DENRLSQKME LLFYMSAVLD SDKLVTYAPS TGGTVGGVGR  250
Neorhizobium (CDN51818.1)          FCNPPSGSSI DEPKLANKME LLFYMSSVLD SDRLVTYAPA TGGSFGGMGQ  250
Sinorhizobium (APG87637.1)         FCNPPAGRRV DENRLSQKME LLFYMSAVMD SDRLVTFVPP TGGSVPGTAR  250
R.phaseoli (ANM08143.1)            FCNPPESRRV DENRLSQKME LLFYMSAVLD SDKLVTYAPS TGGTVGGIGR  248
Agrobacterium (WP_027675286.1)     FCNPPEARRV DENRLSQKME LLFYMDAVLE SDKLVKFASV TSSTTMLL-Q  247
R.ruizarguesonis (NKQ86457.1)      FCNPPESRRV DENRLSRKME LLFYMSAVLD SDKLVTYVPA TGGTVGGMGR  248
R.oryziradicis (WP_075641707)      FWLPPRANRV DENKLAQQME LLFYMTSVLD SDTLVRHAPS IGNGIGGGIG  248
Rhizobium (WP_062451316)           FCNPPQSRRI DENRLSQKME LLFYMDAVLE SDKLVTYAPA SGERAMG--R  246
Sphingomonas (WP_156477649)        IANKPNG-KI DPARLSPKIE LGFYMLWLLD SDELVTQTAP AGPYGGGG--  242
Azospirillum (WP_089286101)        LFKPPVN-EI KRERLTDHIE LSLHMSAMLD SDFLVDIPMH EMSTAGGLGS  243

R.etli Mim1 Re78 (AGS26143.1)      DIEYSKKAIP QLPSAT-DYP KSISPRLTGR PFQPYDPGQR IEYDNIGSVV  299
Neorhizobium (CDN51818.1)          DVEYSRKSIS QMPSNTGHYP KAASPKFVGR PFVPYDPGQR IEYENIGSGI  300
Sinorhizobium (APG87637.1)         ELVLARKPIP QMPSAA-DYP RETAPRLTGK LLQPFEPGTR IEYSNIGSAV  299
R.phaseoli (ANM08143.1)            DIEYGKKAIP QLPSAT-DYP KSISPRLTGR PFQPYDPGQR IEYDNIGSVV  297
Agrobacterium (WP_027675286.1)     DVEFSRTPIA QLPSST-DYP REIGPQSGGI PITSKNIGQR VKYENLGSQI  296
R.ruizarguesonis (NKQ86457.1)      DIEYSKNAIP QLPSAT-DYP KATSPRFIGR PLQPFEPGQR VEYDNIGSVV  297
R.oryziradicis (WP_075641707)      EGIRSKERIN QMPTAK-DYP KESEPKIIRG --ATYEPGQR IEYDDLGSVV  295
Rhizobium (WP_062451316)           DVEYGSKPIS QMPSAA-NYP KAIAPKFTGR PFSPYDPGQR VEYNNIGSAV  295
Sphingomonas (WP_156477649)        --RYSSKPID ALPSSK-DYP R-------GN TDPKRGPIQW VGVTRPGGDV  282
Azospirillum (WP_089286101)        VFGAKERPIP QLPSAS-DYP RGTPFR---T SLGVVHAQQS VRVERPGPIV  289

R.etli Mim1 Re78 (AGS26143.1)      RERINTLSRL TGNSSFYPEQ NS-VEKLFID PTSGAQMVKA EQIINRISAD  348
Neorhizobium (CDN51818.1)          RDRINKLSIS TGNGPFYPNQ SF-VERKLFD PTGHAQMVKA EQIINKLYND  349
Sinorhizobium (APG87637.1)         RERIDTLSRQ YGGSPFYPQQ NF-AEKILID PTGRAQMIRA EQIINRLSAD  348
R.phaseoli (ANM08143.1)            RERINTLSRL TGNSSFYPEQ NS-LEKLFID PTSGAQMAKA EQIINRLSAD  346
Agrobacterium (WP_027675286.1)     RQRIDTLSKL TGNSSFYPEQ SA-VER-FFD PTGNAQLLKA EQIINRLSIE  344
R.ruizarguesonis (NKQ86457.1)      RDRINTLSRL SGNSSFYPEQ NF-AEKLLID PTGSTQLAKA EQIINRLSTE  346
R.oryziradicis (WP_075641707)      RARIDTLSRA VTGSPFYPQQ SIGMRIAGND PTGREQMVKA EHIISRLSAQ  345
Rhizobium (WP_062451316)           RERIDMLSRM TGNSSFYPEQ NF-AERLLID PTGNAQMVKA EQIINRLSAS  344
Sphingomonas (WP_156477649)        EDQIDKVHKQ VRGSAFYAPG GW-FGKSDVN QARLKEVAEA ERVLAWLSDK  331
Azospirillum (WP_089286101)        QTKIDELNGR IFNEKFFSAS SWQGGQNGPM P----ALVRA ETTLNRLAEA  335

R.etli Mim1 Re78 (AGS26143.1)      ARPKQLTDVR MI  360
Neorhizobium (CDN51818.1)          ARPRQLTDVR MI  361
Sinorhizobium (APG87637.1)         ARPKQLVDVR MI  360
R.phaseoli (ANM08143.1)            ARPKQLTDVR MI  358
Agrobacterium (WP_027675286.1)     ARPKELTDVK VI  356
R.ruizarguesonis (NKQ86457.1)      ARPKQLTDVR MI  358
R.oryziradicis (WP_075641707)      TRPKQLSDVF MM  357
Rhizobium (WP_062451316)           ARPKDITDVR MM  356
Sphingomonas (WP_156477649)        TQPLAPLGLR T-  342
Azospirillum (WP_089286101)        SRPLQLDAAR V-  346
```

(b)

```
R.etli Mim1 Re79 (AGS26144.1)    --MLLTGLIK QGFLGRMRWP LAAVTIVGVM AASAAAMVGA SGSTDQSLLQ IEAVSSRLSA  58
Neorhizobium (CDN51817.1)        -MKLISFFSP SVRTGPILRQ SRNVAVAFAM AVWAPAFAAA SDEIDETLLQ IERVGPRLSA  59
Sinorhizobium (APG87638.1)       ---------- ---------- ---------- -----MIAGT SNPIDQSLVQ IEQVNLRLSG  25
R.phaseoli (ANM08142.1)          --MLLTDFLK RGLMGRMRWS LVAAAFTGAM VASAAAMVGA SGSTDQSLLE IEAVSSRLSA  58
Agrobacterium (WP_027675287.1)   MLFNDFTSHV KKICLLKLCA FACLVCVILL TASHVSADQA NNTIDSVVIR AERLDQRLAD  60
R.ruizarguesonis (NKQ86458.1)    ---------- ---MGRMRWP LIAAALVCAM AASAAGMVGA SDTTDQSLLQ IEQVASRLSG  47
R.oryziradicis (WP_175577311.1   ---------- --MIRGLKHS IRGGLLCLGM GMLSPVAACA DPSIDASLVQ IEQVGERVAN  48
Rhizobium (WP_062451254.1)       MMLLVDTVAV LNRVRKVTFL IAAPVLAIML TGCDAGAESS KSSVDASLLR AERVDERLAL  60
Azospirillum (WP_143270660.1)    ---------M RGQELSLLKW SGLLLVLTAL LLAGCSRSGE AEKSDETMMQ MEQVASLYQS  51
Aureimonas (WP_183201854.1)      ---------- ------MRWT TAALALVGVL AAILPGRAMA DPAGDERYLR LEDAERVYAG  44

R.etli Mim1 Re79 (AGS26144.1)    TPAAVETVAK AAADRSVPTS -DAQPLLETA ARSSFDADGM LAEISKAVAE AGSDA-VDPR  116
Neorhizobium (CDN51817.1)        MPSEVENVVK AATDRGNDEA SKAGPILEPV VRASFDPKGM VAEISHSLAE VEGGK-IDPA  118
Sinorhizobium (APG87638.1)       APSAVKTVSK ALAENGNAEV PAAEALLETA VHSSFDSESM GAAILKNIGE VDGGS-IDPE  84
R.phaseoli (ANM08142.1)          TPGAVGSVAK AAADRSVPAS -DAQPLLETA ARSSFDADGM FAEISKAVAE AGSDA-VDPR  116
Agrobacterium (WP_027675287.1)   INAMVENSVA IEAAGTKLDV AKTKPLMLKA ISQKFDLLQM KRTIVAAIEQ PDNDY-QNFK  119
R.ruizarguesonis (NKQ86458.1)    TPAAVENVAK AAADRGISAT SDAQPLLETA ARSSFKVDSM FAEISKAVGE AGDDA-VDPK  106
R.oryziradicis (WP_175577311.1   AGLLADNVLK YISDQEGLHD KQADSMIESA TRDAFNAQAF VTEIQNSMQN VGNSS-IDAQ  107
Rhizobium (WP_062451254.1)       PQALIRNAAS ARRDDKNADI AKTEPLLISS SEKAFDAGEM RKKILVSIEA TPGQY-ADVA  119
Azospirillum (WP_143270660.1)    MPGLVDIVGG DVRKEFGAGV DADLPLLRQS VVKAFDPADL LARVRRELKA VDAGGGAEVP  111
Aureimonas (WP_183201854.1)      MAEFARMQGP LIEVSDRREL PALAERGTAL AAQTFAVAGA RDAMLERLRK TPGEV---PV  101

R.etli Mim1 Re79 (AGS26144.1)    AFAKAAAAWH EGQAKVARMY QAQDQVAAKE IETRLADPDK GPGIKALADL MAGPDLAVES  176
Neorhizobium (CDN51817.1)        TLAKAAAAFE DGRKKIAEMY RSQDQAIAKQ IEARIAGADD GPRIKQLADL MASPELAAET  178
Sinorhizobium (APG87638.1)       AFARAAAAFE EGRGKIARIY EAQDQTAAKE IEARLADPET GARLRQLTDL MAAPELAVES  144
R.phaseoli (ANM08142.1)          AFAKAAAAWH EGQAKVARMY QAQDQVAAKE IETRLADPDK GPGIKALADL MAGPDLAVES  176
Agrobacterium (WP_027675287.1)   ALDKAVSALI VSYAQIDKIY TDKDEVAGKS IQARLGNPKT RETVNDVANL MASPKLAADA  179
R.ruizarguesonis (NKQ86458.1)    AFAKVAAALE AGQAKIARMY EAQDQTAAKD IEMRLANPDN GPRISQLADL MAGPDLAVDT  166
R.oryziradicis (WP_175577311.1   RLAKIADDLN ASRKKIETLA QSKGDAALQT MDAEIVKKAD SPRVEQLAQL MASPDLAAET  167
Rhizobium (WP_062451254.1)       AFDTATAALA DGYRAVDKMY ADKDEDFGKK IETRLASPEA KSTIEEVASL MASPDLAAET  179
Azospirillum (WP_143270660.1)    AFLAAANDFI RTQSAVEAES RVDPGKVIQS ATDAYEVRAD KGRIDALAGA MAAADIQVEG  171
Aureimonas (WP_183201854.1)      AFDAIVARMR AVEAEFASRS LEALQAGEAD AVAHYEARPD RASIDAVGDA MAAPDLARET  161

R.etli Mim1 Re79 (AGS26144.1)    AVTWQTMYLL LEALSSADAE KLASVSQDKL QSEMNGVIAS LRAR---SEN EKPLPKDMAR  233
Neorhizobium (CDN51817.1)        ALTAQVMYVS LEAFSDPNSA ELASASSEKR KSELGGVIAS LRDR---TEN EKPVPRDIAR  235
Sinorhizobium (APG87638.1)       AFTSQLMYAA MEAFSDPSAA ELASSS---- --ETPHVIAS LRSR---NEN EKPIAKDAAR  195
R.phaseoli (ANM08142.1)          AVTWQTMYLS LEALSSADAE KLASVSQDKL QSEMNGVIAS LRAR---SEN EKPLPKDMAR  233
Agrobacterium (WP_027675287.1)   ASFQQAVGVA LKISSDPGIY ELNEEDQKKF LADLPNFLAN LRER---EVN QSAYSRVITE  236
R.ruizarguesonis (NKQ86458.1)    AFTAQLMYLC LEALSNSDVE TLASASKDKL QSEMQGVISS LRSK---SEN EKPVPKDIAR  223
R.oryziradicis (WP_175577311.1   AVTAQAMYVA VHAFFNSDPS QFAALSQEQR QDMPTQILSY LQQK---TEN EKPYSKDANR  224
Rhizobium (WP_062451254.1)       AVTQQAISAV LTMETGSPAL DPSSEEHKQL KATLPSLLPE FRNR---PDQ GGAYSKESAK  236
Azospirillum (WP_143270660.1)    TLLGLRLQGA AALRHLEKPV LWQGLDDQGR DRYADQFTRR TRAEG--DAN HSPPRYEIAR  229
Aureimonas (WP_183201854.1)      AVTGLRVKWI YETLLTGNMD EMRAMTPALV ETGIREVLDR TRAPDDPEYG APPLRKELVR  221

R.etli Mim1 Re79 (AGS26144.1)    ADEKARLTFV LATMPPEDLS VLTEFYESSG GKAKRDALVE SYRRVSDQAN KQMLGQYFQA  293
Neorhizobium (CDN51817.1)        MEEKARLTFV LAALPKDDLS VLSDFYQSAE GKAKRQALVE TYRQVSDQAN TRMLEKYFSA  295
Sinorhizobium (APG87638.1)       LEEQGQLSFI LATLSYEDLS VLLDFYRSSG GKTKRQALVD SYVKVSDQAN TKMLQAYFSA  255
R.phaseoli (ANM08142.1)          ADEKARLTFV LATMPPEDLS VLTEFYESSG GKAKRDALVE SYRRVSDQAN KQMLGQYFQA  293
Agrobacterium (WP_027675287.1)   EYARNELSYA LATLSDDQLD TLKMFYSSPT GIAKTEALKK TYKDILSENT SIALDQYFRL  296
R.ruizarguesonis (NKQ86458.1)    ANEKTRLTFV LATLSPEDVS VLTDFYESPG GKAKRDALIA SYRQASDQAN TEMLGQYFQA  283
R.oryziradicis (WP_175577311.1   ANEKLRLGLA LAGLSDGDIL YLTNLYQSPE GKAKRAALLD RYKRLSTDAN QKMLQDYFTQ  284
Rhizobium (WP_062451254.1)       AFETAGLTFA LATLTDKDLS ALKSFYAGSY GKSKRQELLG AYQQVWDAAA TTMLTTYIEE  296
Azospirillum (WP_143270660.1)    FLAIQRLTII LLRLPEPSLK ALEDFYNSDL GKAKRKALVA AVAAQSDADT KQMMVEYLRQ  289
Aureimonas (WP_183201854.1)      EQARMVKRMV LSHLPEADVA ALAAFYASDV GRAKRRALVE AFGARNDEDG ARFIRQLLER  281

R.etli Mim1 Re79 (AGS26144.1)    LAEYFKTHPR PQQQQ 308
Neorhizobium (CDN51817.1)        LAGYFKTHPR PQQQ- 309
Sinorhizobium (APG87638.1)       LANYLKTHPR PQQQ- 269
R.phaseoli (ANM08142.1)          LAEYFKTHPR PQQQQ 308
Agrobacterium (WP_027675287.1)   IFAEGVKN-- ----- 304
R.ruizarguesonis (NKQ86458.1)    LADYLKTHPR PQQQQ 298
R.oryziradicis (WP_175577311.1   LADNLTKRKS NN--- 296
Rhizobium (WP_062451254.1)       LAKQHPAAVK AQ--- 308
Azospirillum (WP_143270660.1)    LR-------- ----- 291
Aureimonas (WP_183201854.1)      ERSRTP---- ----- 287
```

**Figure S2.** Complete alignment of Re78 and Re79 with homologous proteins. (**a**) Re78 (ReMim1 AGS26143.1) alignment showed 39 identical amino acids (red). (**b**) Re79 (ReMim1 AGS26144.1) alignment presented 11 identical amino acid (red). The amino acid position of residues is indicated on the right side of the sequences. The NCBI ID is shown on the left of each sequence.

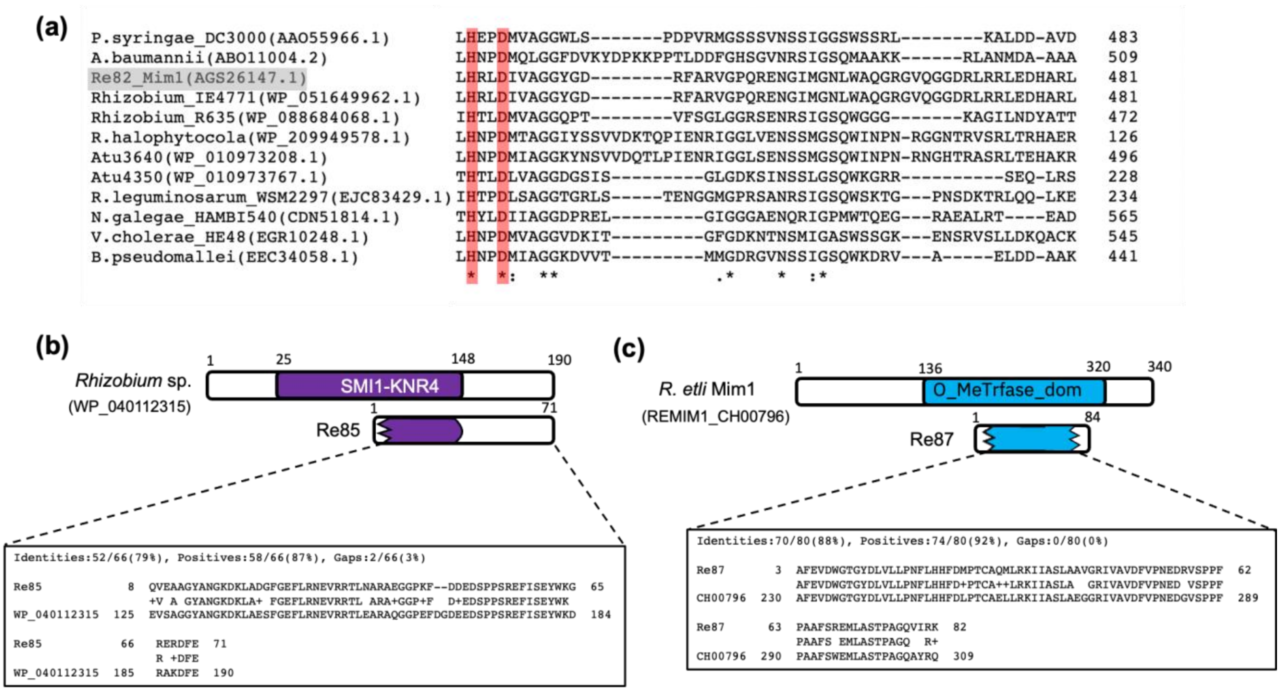


**Figure S3.** Relevant characteristics of the Re82, Re85 and Re87 proteins encoded in the T6SS cluster of ReMim1. (**a**) Partial sequence alignment of Re82 and members of a superfamily of T6SS-dependent DNase effectors. The HxxD catalytic motif is highlighted in red. Asterisks and dots indicate conserved amino acid and conservative changes, respectively. The amino acid position of residues shown is indicated on the right of the sequences. The NCBI ID are shown on the left of each sequence (**b**) Alignment of protein Re85 with a representative of a BLAST search, *Rhizobium* sp. (WP_040112315). SMI1-KNR4 domain is highlighter in purple. (**c**) Alignment of protein Re87 with a representative of a BLAST search, *Rhizobium etli* Mim1 (REMIM1_CH00796). O-methyltransferase domain is highlighted in blue. Organism names and protein accession numbers are on the left and the amino acid position in the alignments is indicated on each side of the sequences.

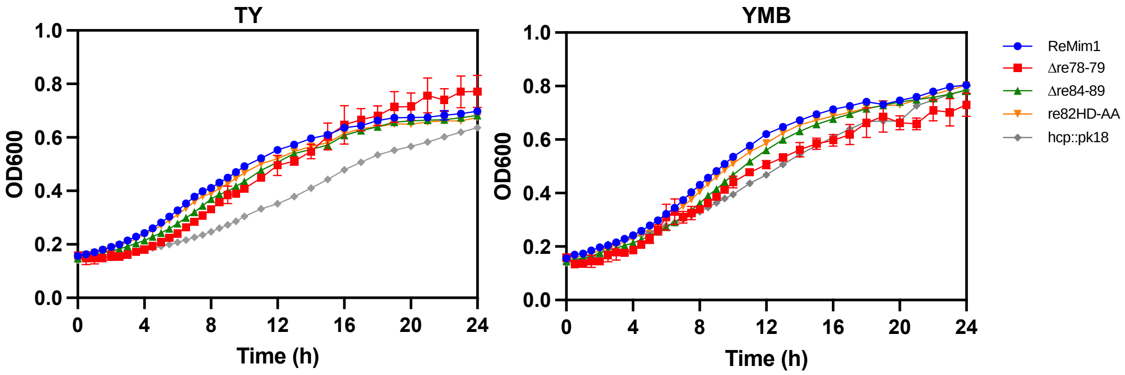


**Figure S4.** Growth curves of *Rhizobium etli* Mim1 strain and T6SS-derivative mutants in TY and YMB media at 28 °C.

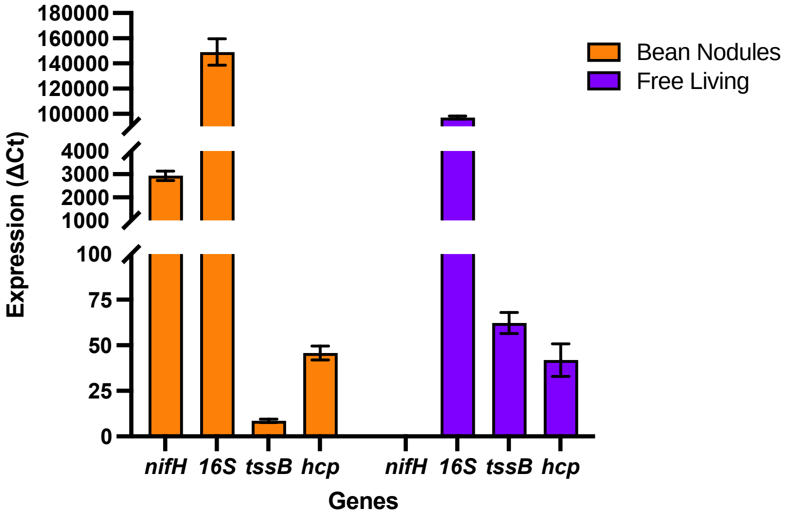


**Figure S5.** RT-qPCR analysis of T6SS genes *tssB*, *hcp* and*16S rRNA* gene (constitutive) and *nifH* gene (expressed in symbiotic condition). RNA from *tssB*, *hcp* and 16S rRNA were analysed in free-living cells (TY medium) and RNA from *tssB*, *hcp* and *nifH* were analysed from bean nodules induced by *Rhizobium etli* Mim1, 28 dpi. Data were normalized using expression of *rpoD* gene. Bars values are average of two biological assays with three replicates.

**Table S1.** Bacterial strains and plasmids, related to Experimental Procedures.

| Strain or Plasmid | Relevance Characteristics | Reference or Source |
|---|---|---|
| *Escherichia coli* | | |
| DH5α | Host for DNA cloning | Gibco-BRL |
| BL21(DE3) | Host for protein expression | New England Biolabs |
| S17.1 | Host for conjugation | [72] |
| HB101 | Host for conjugation, triparental mating | [73] |
| *Rhizobium* | | |
| *Rhizobium etil* Mim1 (ReMim1) | Wild-type strain able to induce nodules in *Phaseolus vulgaris* plants | [74] |
| hcp::pk18 | ReMim1 *hcp* mutant, $Km^r$ | [21] |
| Δre78-79 | ReMim1 *re78* and *re79* deletion mutant | This work |
| re82HD-AA | ReMim1 derivative by site-directed mutagenesis in amino acids H431 and D434 has been substituted for Alanines | This work |
| Δre84-89 | ReMim1 derivative with *re84* to *re89* genes deleted | This work |
| Plasmids | | |
| pCR2.1-TOPO | Vector for optimize cloning PCR products, $Amp^r$, $Km^r$ | Invitrogen |
| TOPO.Re79StrepTag | pCR2.1-TOPO derivative containing *re79* gene | This work |
| pCR2.1-P6 | pCR2.1-TOPO derivative containing P6 region (1242-bp) of T6SS from ReMim1 | This work |
| pSCA | Vector for optimize cloning PCR products, $Amp^r$, $Km^r$ | Agilent Technologies |
| pSCA-re82 | pSCA derivative containing *re82* gene | This work |
| pSCA-HD→A | pSCA derivative containing *re*82 $H_{431}A$ | This work |
| pSCA-AD→A | pSCA derivative containing *re*82 $H_{431}A$-$D_{434}A$ | This work |
| pK18*mobsacB* (pK18) | Suicide vector; *sacB* gene for double crossover event selection, $Km^r$ | [75] |
| pK18*mobsacB*.Δre78-79 | pK18 derivative to generate a *re78-79* deletion | This work |
| pK18*mobsacB*.Δre84-89 | pK18 derivative to generate a re84-89 deletion | This work |
| pK18*mobsacB*.re82HDAA | pK18 derivative to generate *re*82 $H_{431}A$-$D_{434}A$ | This work |
| pET22b(+) | T7 promoter vector with N-terminal signal peptide (PelB), $Amp^r$ | Novagen |
| pET22b.Re78SP | pET22b derivative expressing Re78 with a N-terminal PelB sequence | This work |
| pET22b.Re78 | pET22b derivative expressing Re78 | This work |
| pBAD33 | Plasmid for cloning, L-arabinose inducible, araBAD promoter, $Cm^r$ | [76] |
| pBAD33.Re79 | pBAD33 expressing immunity protein Re79, $Cm^r$ | This work |
| pLMB509 | Broad-host range expression vector with a taurine inducible promoter, $Gm^r$ | [77] |
| pLMB509-Re79StrepTag | pLMB509 derivative expressing Re79 with a C-terminal Strep-tag | This work |
| pMP220 | Cloning vector with a promoter-less *lac*Z gene, $Tc^r$ | [78] |
| pMP220-PTssA | pMP220 cloned P6 of ReMim1 and *tss*A region, $Tc^r$ | This work |
| pMP220-PHcp | pMP220 cloned P6 of Remim1 and *tss*H region, $Tc^r$ | This work |
| pHC60 | Green Fluorescent Protein (GFP) gene under a constitutive promoter, $Tc^r$ | [79] |
| pCMB13 | Contains the *Discosoma* Red fluorescent protein (DsRed) gene under a constitutive promoter, $Sp^r$ | [80] |

Antibiotic Abbreviations: Amp (ampicillin), Km (kanamycin), Sp (spectinomycin), Tc (tetracycline), Gm (gentamicin), Cm (chloramphenicol).

**Table S2.** Primers used in this study, related to experimental procedures.

| Primer | Sequence (5 '-3') | Use |
|---|---|---|
| **P78.F** | CCGGCCATATGATCGTGTCGGCTGCAACTGA | Cloning of *re78* in pET22b without PelB |
| **P78.R** | CCGGGCTCGAGTTAATGGTGATGGTGATGGTGAATCATCCTGACATC | |
| **P78sp.F** | CCGGGGGATCCGTCGGCTGCAACTGAGCGGCA | Cloning of *re78* with PelB (N-terminal) |
| **P78sp.R** | CCGGGCTCGAGTTAATGGTGATGGTGATGGTGAATCATCCTGACATC | |
| **P79.F** | CCGGCCATATGCTGTTGACCGGTCTTATCAAG | Cloning of *re79* in pBAD33 |
| **P79.R** | CCGGCAGCGCTTTAATGGTGATGGTGATGGTGCTGCTGCTGTTGCGGCCGGGG | |
| **P79.NdeI** | CCGGGCATATGCTGTTGACCGGTCTTATC | Cloning of *re79* in PCR2.1 TOPO and pLMB509 |
| **P79.NdeIStrep** | CCCGGCATATGTTACTTTTCGAACTGCGGGTGGCTCCAGCTAGCCTGCTGCTGTTGCGG | |
| **P78.1** | CCTCACCAAGGTCAGCGATT | Deletion of *re78* to *re79* |
| **P78.2** | CACCAAACGTGGCTTGCCGTACTTGGGGAGTTCTACCAGA | |
| **P79.3** | GGCAAGCCACGTTTGGTGGCGCTGGCGGAGTATTTCAA | |
| **P79.4** | CGACCGAGGTCTTGTCCATC | |
| **P84.1** | GCGAAAACGGGATTATGGGC | Deletion of *re84* to *re89* |
| **P84.2** | ACCCGCTTCATAGCGATCATCGGTCCAACG | |
| **P89.1** | TGATCGCTATGAAGCGGGTCGATTAGGAGG | |
| **P89.1** | GCTGGCGCGCAATACAATTA | |
| **P82.F** | GATTGGCAGGCTAGGCAGAT | Site-directed mutagenesis in which amino acids $H_{431}$ and $D_{434}$ have been substituted for Alanines |
| **P82.R** | TGAAGACGGGATTGTAGGCG | |
| **PH.A.F** | CTGGCCGCACTGGCACGCCTTGATATT | |
| **PH.A.R** | AATATCAAGGCGTGCCAGTGCGGCCAG | |
| **PD.A.F** | TGGCACGCCTTGCTATTGTGGCGGG | |
| **PD.A.R** | CCCGCCACAATAGCAAGGCGTGCCA | |
| **pMP220 F** | AGCTCCTGAAAATCTCGTCG | Confirmation of P6 orientation in pMP220 |
| **pMP220 R** | TAAAAACCCCACTCCGGCAA | |
| **P6.1** | GTCGAGCATTGCCCGGT | Amplification of ReMim1 P6 promoter region |
| **P6.2** | CCGACATCCGTGCGACA | |
| **78.RT.F** | GAGCGTTTGAACGATATTCT | qPCR of T6SS genes |
| **78.RT.R** | CTGACATAGGGACTGGAAAC | |
| **79.RT.F** | TGACGGAATTCTATGAGAGC | |
| **79.RT.R** | GGGATGGGTCTTGAAATACT | |
| **81.RT.F** | TTCTGCCAGAGAACCCAAAC | |
| **81.RT.R** | CCATAGGCCTTACTCCAGCA | |
| **82.RT.F** | GACGTAGATGATGTCGAGGA | |
| **82.RT.R** | ACTGGTTCTTCTCTCGGTCT | |
| **83.RT.F** | AGTGCTCTCGGACCTCTGAA | |
| **83.RT.R** | CTCGACCCCTATACGGACAA | |
| **84.RT.F** | CTATTTCCGAGACAGGAATG | |
| **84.RT.R** | GACTTCAACTGCGAATGAAT | |
| **85.RT.F** | GTTCCTGCGCAACGAAGT | |
| **85.RT.R** | GTTCCCGACCCTTCCAGTAT | |
| **86.RT.F** | GGCAGTGGGTCTGGACATAG | |
| **86.RT.R** | GATACGACTGCGGAGCACAC | |
| **87.RT.F** | CTGCCTTCGAGGTAGATTGG | |
| **87.RT.R** | CGATGATCTTTCGGAGCATT | |
| **88.RT.F** | ACCGCACGCTATCAGCTC | |
| **88.RT.R** | GTCGAGAACCGGCTTCAG | |
| **89.RT.F** | GCTGCCGGATCATAGTCAAA | |
| **89.RT.R** | GGCAGCAAGGTTTGAAAAGT | |

| | |
|---|---|
| **hcp.RT.F** | TTTCGGAAGTGATCCTCACC |
| **hcp.RT.R** | ATATAGGTGTCGCCGGGATT |
| **nifH.RT.F** | GGTCTTCATGATGCCGAAGT |
| **nifH.RT.R** | CTAGCCGAGAAGATCCATGC |
| **tssB.RT.F** | AAGCTCCGTTCCGTCTTTCT |
| **tssB.RT.R** | ATCCGACACAGCCTCTCAAG |
| **16S.RT.F** | ACGTGCTACAATGGTGGTGA |
| **16S.RT.R** | CAGAGTGCAATCCGAACTGA |
| **rpoD.RT.F** | GATGAAGTCGATCGGCAATCTG |
| **rpoD.RT.R** | GCTTCGACCATTTCCTTCTTGG |

**Table S3.** Proteomic analysis of Re78/79 from secretomes of *Rhizobium etli* Mim1 and hcp::pk18 mutant.

| **Protein** | **Accession** | **MW (kDa)** | **SignalP** [a] | **Score** [b] | | **Peptides** [c] | | **PSM** [d] | |
|---|---|---|---|---|---|---|---|---|---|
| | | | | **ReMim1** | **hcp::pk18** | **ReMim1** | **hcp::pk18** | **ReMim1** | **hcp::pk18** |
| Re78 | S5S7Y0 | 39.5 | No | 160.97 | 11.68 | 18 | 2 | 54 | 4 |
| Re79 | S5SXD8 | 32.8 | Yes | 42.25 | 35.21 | 6 | 6 | 14 | 13 |

[a] Signalpeptide identified by SignalP 5.0; [b] Measure of the goodness of fit of experimental peptide fragments to theoretical spectra; [c] Total number of distinct peptide sequences identified; [d] Relative Peptide-Spectrum-Match.
